# A Small Molecule Inhibitor of Sarcomere Contractility Acutely Relieves Left Ventricular Outflow Tract Obstruction in Feline Hypertrophic Cardiomyopathy

**DOI:** 10.1371/journal.pone.0168407

**Published:** 2016-12-14

**Authors:** Joshua A. Stern, Svetlana Markova, Yu Ueda, Jae B. Kim, Peter J. Pascoe, Marc J. Evanchik, Eric M. Green, Samantha P. Harris

**Affiliations:** 1 Department of Medicine & Epidemiology, School of Veterinary Medicine, University of California Davis, Davis, California, United States of America; 2 MyoKardia, Inc., South San Francisco, California, United States of America; 3 Department of Surgical & Radiological Sciences, School of Veterinary Medicine, University of California Davis, Davis, California, United States of America; 4 Department of Cellular and Molecular Medicine, College of Medicine, University of Arizona, Tucson, Arizona, United States of America; University of Cincinnati, UNITED STATES

## Abstract

Hypertrophic cardiomyopathy (HCM) is an inherited disease of the heart muscle characterized by otherwise unexplained thickening of the left ventricle. Left ventricular outflow tract (LVOT) obstruction is present in approximately two-thirds of patients and substantially increases the risk of disease complications. Invasive treatment with septal myectomy or alcohol septal ablation can improve symptoms and functional status, but currently available drugs for reducing obstruction have pleiotropic effects and variable therapeutic responses. New medical treatments with more targeted pharmacology are needed, but the lack of preclinical animal models for HCM with LVOT obstruction has limited their development. HCM is a common cause of heart failure in cats, and a subset exhibit systolic anterior motion of the mitral valve leading to LVOT obstruction. MYK-461 is a recently-described, mechanistically novel small molecule that acts at the sarcomere to specifically inhibit contractility that has been proposed as a treatment for HCM. Here, we use MYK-461 to test whether direct reduction in contractility is sufficient to relieve LVOT obstruction in feline HCM. We evaluated mixed-breed cats in a research colony derived from a Maine Coon/mixed-breed founder with naturally-occurring HCM. By echocardiography, we identified five cats that developed systolic anterior motion of the mitral valve and LVOT obstruction both at rest and under anesthesia when provoked with an adrenergic agonist. An IV MYK-461 infusion and echocardiography protocol was developed to serially assess contractility and LVOT gradient at multiple MYK-461 concentrations. Treatment with MYK-461 reduced contractility, eliminated systolic anterior motion of the mitral valve and relieved LVOT pressure gradients in an exposure-dependent manner. Our findings provide proof of principle that acute reduction in contractility with MYK-461 is sufficient to relieve LVOT obstruction. Further, these studies suggest that feline HCM will be a valuable translational model for the study of disease pathology, particularly LVOT obstruction.

## Introduction

Hypertrophic cardiomyopathy (HCM) is a heritable disease of the heart muscle characterized by asymmetric thickening of the left ventricular walls not explained by another cardiac or systemic disease. It is the most common heritable heart disease in humans with a prevalence of one in 500 individuals [1]. Heart function in patients with HCM is characterized by hyperdynamic contraction [2] and impaired relaxation [3]. The clinical course is variable with some patients experiencing minimal symptoms and others developing exertional dyspnea, heart failure, atrial fibrillation and stroke, or sudden cardiac death [4].

A predictor of adverse outcomes in HCM is the presence of left ventricular outflow tract (LVOT) obstruction [5]. This phenomenon occurs when one or both of the mitral valve leaflets contact the interventricular septum during systole (systolic anterior motion or SAM) and impede bloodflow into the LVOT. As a result, a pressure gradient develops between the ventricle and aorta, ventricular hypertrophy worsens and patients more frequently become symptomatic. Although recognized as an important feature of HCM, the detailed mechanism of SAM, and the relative contribution of distorted ventricular geometry, hypercontractility and structural abnormalities of the mitral valve to generation of LVOT obstruction remain active areas of investigation [6].

Approximately one-third of patients have LVOT obstruction at rest, and an additional one-third develop obstruction only after provocation with exercise or other hemodynamic stressors [7]. When this pathology is accompanied by persistent heart failure symptoms despite conventional therapies, patients may be eligible for invasive septal reduction therapy. Patients with severely obstructive (≥50 mmHg gradient), persistently symptomatic HCM who undergo invasive septal reduction therapy (surgical myectomy or alcohol septal ablation) experience improvement in symptoms, exercise capacity and prognosis in proportion to the resulting LVOT gradient [8,9]. However, these interventions do not address the underlying molecular pathology of disease. Currently available medical therapies for reducing obstruction (beta blockers, calcium channel blockers and disopyramide) act through non-specific mechanisms, achieve variable therapeutic responses and have pleiotropic pharmacological properties (effecting inotropy, chronotropy, cardiac conduction, and vascular tone) that limit tolerability. None of these pharmacologic therapies are demonstrated to improve the symptoms and functional limitations associated with LVOT obstruction or to modify the natural history of disease [10]. New therapies with more targeted pharmacology are needed and could provide a medical alternative to invasive therapies.

The development of new treatments has been limited in part by a lack of preclinical models for HCM with obstructive physiology. Mouse models of HCM have been crucial in advancing understanding of the biology and molecular biophysics of disease [11], but none develop SAM or LVOT obstruction. In cats, HCM is recognized as a frequent cause of heart failure, disability and death [12]. In a study of an unselected population of cats in a shelter, ~15% met criteria for HCM [13]. Cats with HCM exhibit many structural and clinical features of the human disease, including myocyte hypertrophy, myofibrillar disarray, and fibrosis, as well as chronic heart failure, an elevated risk for thromboembolism and sudden cardiac death [14]. There is also a shared molecular mechanism, with two mutations in *MYBPC3* (the most commonly mutated gene in human HCM) [15] implicated as disease-causing in cats [16]. LVOT obstruction caused by SAM is a feature of some feline HCM, although its prognostic value has not been evaluated in a prospective study [17]. Cats may thus represent an appealing large-animal system to study HCM pathophysiology—particularly LVOT obstruction—and potential therapeutic interventions.

Studies of tissues and purified proteins bearing mutations causing HCM are largely consistent with the idea that HCM arises from a hypercontractile sarcomere [18]. Building on these insights, a small molecule, MYK-461, that directly inhibits sarcomere force output to reduce contractility was recently identified [19]. In contrast to existing medical therapies for HCM, MYK-461 acts directly on myosin, the force generating protein in muscle and molecular source of disease. In mouse models of HCM, early treatment with this molecule attenuates ventricular hypertrophy, myofibrillar disarray and fibrosis, suggesting that reducing contractility at the sarcomere can modify the characteristic cellular and structural pathology of HCM. However, it remains uncertain whether reducing sarcomere contractility is sufficient to correct already established hemodynamic abnormalities of HCM including, importantly, LVOT obstruction. Here, we describe studies in cats with HCM and dynamic LVOT obstruction to test the hypothesis that a targeted reduction in contractility with a small molecule can acutely eliminate SAM and reduce LVOT obstruction.

## Methods

MYK-461 was synthesized at MyoKardia, Inc. For feline studies, 0.25 mg/kg MYK-461 was prepared as sterile solution in PEG400/saline at 1/2 ratio for intravenous administration.

### Animals

Care and handling of all animals was in accordance with the National Research Council Guide for the Care and Use of Laboratory Animals using protocols approved by the Institutional Animal Care and Use Committee at University of California, Davis. Cats were obtained from a research colony of Maine Coon/mixed breed cats with heritable HCM [14,20,21].

### Experimental protocol

Five cats were selected for study that met previously established criteria [13] for feline HCM and exhibited LVOT obstruction at rest under butorphanol and acepromazine sedation. All cats received a combination of alfaxalone and midazolam for anesthetic induction, followed by inhaled isoflurane and oxygen maintenance. Blood pressure was measured via an automated blood pressure cuff at five-minute intervals with supplementary continuous monitoring by arterial line when arterial access could be obtained. Following induction of anesthesia, a complete baseline echocardiogram was performed (timepoint #1). Cats were then treated with atropine 0.04 mg/kg IV, followed by an infusion of isoproterenol 0.04 μg/kg/min IV to elevate heart rate back to pre-anesthetized values and induce the previously observed LVOT obstruction. After five to seven minutes, a stable heart rate of 200–220 bpm was reached and a complete echocardiogram was performed (timepoint #2). At the completion of imaging, a ten-minute intravenous infusion of MYK-461 (n = 5) at 0.3 mg/kg/hr IV was started (S1 Fig). Focused echocardiography was performed after five minutes (timepoint #3). After ten minutes, the MYK-461 infusion rate was lowered to 0.12 mg/kg/hr IV, a blood sample was drawn and an echocardiogram performed (timepoint #4). If ventricular function remained hypercontractile or within normal limits by visual inspection, another blood sample was obtained and the MYK-461infusion rate was increased to 0.36 mg/kg/hr IV for ten minutes. Focused echocardiography was performed after five minutes (timepoint #5). After ten minutes, the MYK-461 infusion rate was lowered to 0.15 mg/kg/hr IV, a blood sample was drawn and an echocardiogram performed (timepoint #6). Following imaging, the isoproterenol infusion was discontinued. When heart rate returned to baseline levels (five to seven minutes), a complete echocardiogram was performed on MYK-461 alone (timepoint #7). Study drug was then discontinued, and animals were awakened, extubated and moved to recovery. Three of five cats were available to return for a control arm of this experiment after a 6-week washout period. The experiment was completed with identical methodology with the exception that MYK-461 was substituted for vehicle (control) at matched volumes/infusion rates.

### Echocardiography

Complete two-dimensional, m-mode, color and spectral Doppler echocardiography was performed from standard imaging planes in right and left lateral recumbency as previously described [22]. Briefly, assessment of left ventricular hypertrophy was made from either m-mode imaging in the right parasternal short axis view at the level of the papillary muscles or two-dimensional imaging in the short or long axis plane at the same level. Segmental LV wall measures in diastole that exceeded 6mm in the absence of other disease (systemic hypertension, hyperthyroidism) were considered consistent with HCM. Color flow and spectral Doppler as well as m-mode imaging was utilized to evaluate for systolic anterior motion of the mitral valve and measure LVOT velocity as a surrogate marker of pressure gradient.

The initial echocardiogram for evaluating inclusion criteria was performed on awake, sedated (butorphanol 0.25mg/kg and acepromazine 0.05mg/kg intramuscularly) cats.

Once selected and enrolled in this study, all cats were anesthetized for the remaining echocardigoraphic assessments. Anesthesia was performed with alfaxalone and midazolam premedication/induction then cats were intubated and maintained on inhaled isoflurane and oxygen. A stable anesthetic plane was achieved prior to any drug intervention.

Focused echocardiography was obtained from the right parasternal short and long axis imaging planes, where wall and chamber measures were obtained by both two-dimensional and m-mode methods. Subcostal imaging was performed to provide superior alignment with the left ventricular outflow tract and assess the left ventricular pressure gradient throughout the study by continuous wave spectral Doppler.

### MYK-461 Pharmacokinetics

MYK-461 pharmacokinetics was established in felines (n = 3) after a single dose IV bolus injection. Blood samples were collected 0.083, 0.25, 0.5, 1, 2, 4, 8, 24, 48, 72 and 336 hours post-dose into K_2_-EDTA collection tubes. Plasma concentrations of MYK-461 were measured after protein precipitation using a qualified LC-MS/MS method on AB Sciex Q-Trap API 5500 equipped with Thermo Fisher Scientific LC Aria LX2 with diverter valve. Samples were separated on Kinetex 5u C18 100Å 30 x 2.1 mm analytical column (Phenomenex, Torrance, CA), with gradient mobile phases consisting of 0.1% formic acid in water and 0.1% formic acid in acetonitrile. Sample concentrations were determined from weighted linear regressions of peak area ratios (peak areas of MYK-461 divided by peak areas of internal standard) versus nominal concentrations of the calibration curve standards. All calibration standards and quality control samples met ±15% accuracy acceptance criteria. The assay had a lower limit of quantitation of 2 ng/mL. Phoenix WinNonlin Software (Certara, Princeton, NJ) was used to calculate pharmacokinetc parameters, model and establish infusion protocol for pharmacodynamic study to reach target pharmacodynamic effect.

## Results

A research colony of mixed breed (domestic short hair) cats with HCM was generated from a single Maine Coon/mixed breed founder cat presenting with naturally-occurring disease [14,21]. To identify cats in this colony with LVOT obstruction, we used echocardiography in conscious animals to document hypertrophy (interventricular septum or left ventricular posterior wall [diastole] > 0.6 cm) and blood flow acceleration in the LVOT consistent with obstruction by continuous wave spectral Doppler, color Doppler, 2D and M-mode imaging (S2 Fig). Five male cats ranging in age from 0.9 to 3.7 years met these criteria (Table 1). Three of these cats exhibited more diffusely distributed hypertrophy with both left ventricular posterior wall and interventricular septum measures (diastole) > 0.5 cm. The remaining two cats had a more segmental pattern of hypertrophy with disproportionate thickening of the interventricular septum. Four cats demonstrated hyperdynamic left ventricular function (fractional shortening (FS) > 55%), while one cat was within the normal range (35% < FS < 55%), consistent with prior findings in both feline [17] and human HCM [23]. Three cats had enlarged left atria (left atrial diameter / aortic root diameter > 1.5), indicative of chronically elevated left ventricular filling pressures. These structural and functional parameters show that cats selected for this study have morphologic signs of HCM with LVOT obstruction and chronic hemodynamic abnormalities.

**Table 1:** Baseline demographic and echocardiographic characteristics.

ID	Age (years)	Sex	IVSd (cm)	LVPWd (cm)	LVd (cm)	LVs (cm)	FS (%)	LA/Ao
2011–26	3.7	M	0.66	0.47	0.98	0.43	56.4	1.35
2011–25	3.7	M	0.66	0.50	1.59	0.52	67.5	1.55
2011–17	3.9	M	0.72	0.57	1.13	0.39	65.6	1.52
2011–31	2.8	M	0.59	0.63	1.55	0.57	63.4	1.46
2011–44	0.9	M	0.63	0.60	1.27	0.76	40.6	1.53
Normal			<0.60	<0.60			35–55	<1.5

**Fig 1:**
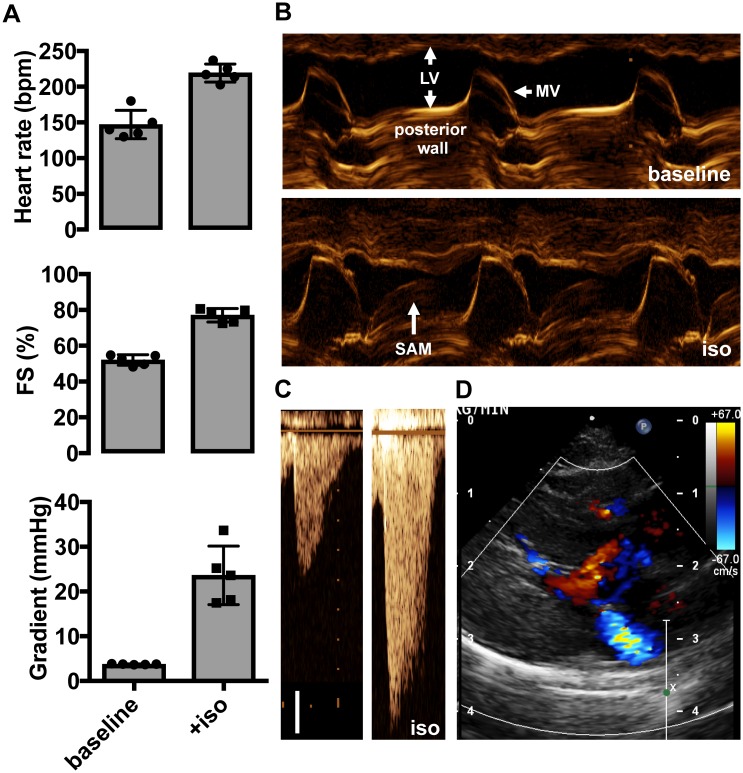
Isoproterenol increases heart rate and contractility and induces dynamic LVOT obstruction in cats with HCM. Treatment with isoproterenol 0.04 μg/kg/min IV **(A)** augments heart rate, fractional shortening (FS) and LVOT gradient (n = 5, mean ± SD), **(B)** provokes mitral valve SAM and mitral/septal contact (LV = left ventricle cavity, MV = anterior leaflet mitral valve), **(C)** induces an LVOT pressure gradient (scale bar 0.5 m/s) and **(D)** mitral regurgitation**.**

To characterize the dynamics of ventricular blood flow and outflow tract obstruction in more detail, we performed complete echocardiograms in this cohort under anesthesia. Anesthesia was necessary to accurately measure LVOT velocities from subcostal views poorly tolerated by conscious cats and to maintain consistent hemodynamic parameters, but did alter resting hemodynamics. During the baseline echo, heart rates in conscious cats ranged from 200–250 beats per minute (bpm). Anesthetized cats had slower heart rates (147 ± 20 bpm), which were accompanied by a decrease in FS (52 ± 3%) (Fig 1A). Under these conditions, LVOT obstruction and SAM were obliterated (Fig 1A–1C). From the earliest descriptions of HCM, LVOT obstruction has been recognized as dynamic and often “intensified” by adrenergic agonists [24]. To reproduce the LVOT obstruction observed in conscious animals, anesthetized cats were therefore treated with isoproterenol 0.04 mcg/kg/min IV, which elevated heart rates to levels comparable to the conscious state (from 147 ± 20 bpm to 219 ± 13 bpm) and increased FS (from 52 ± 3% to 77 ± 4%) (Fig 1A, S3 Fig). All five cats under study developed SAM and evidence of LVOT obstruction (Fig 1A–1C). In one cat, SAM was accompanied by a mild posteriorly-directed jet of mitral regurgitation (Fig 1D). These data indicate that this cohort of cats has dynamic SAM and LVOT obstruction dependent on heart rate and contractility.

Because conventional negative inotropes also directly modulate heart rate and/or vascular tone, it has not been possible to pharmacologically isolate the contribution of changes in contractility to the magnitude of LVOT obstruction. To address this question, we treated cats with MYK-461, a recently-described, mechanistically novel small molecule that inhibits sarcomere contractility by directly binding β-cardiac myosin heavy chain and reducing its ability to productively engage the thin filament [19]. Studies of MYK-461 in animals and isolated muscle-fiber preparations have demonstrated concentration-dependent reductions in contractility without changes to the intracellular calcium transient.

To observe regulation of contractility by MYK-461 in the feline heart, we administered MYK-461 as an intravenous infusion and monitored heart function by echocardiography. Compound infusion rates were calculated from dose-ranging pharmacokinetic studies of MYK-461 to target up to 25% relative reduction in FS (see Detailed Methods). In three cats available for follow up, studies were repeated with an infusion of vehicle alone after a washout period of six weeks to ensure complete elimination of MYK-461.

**Fig 2:**
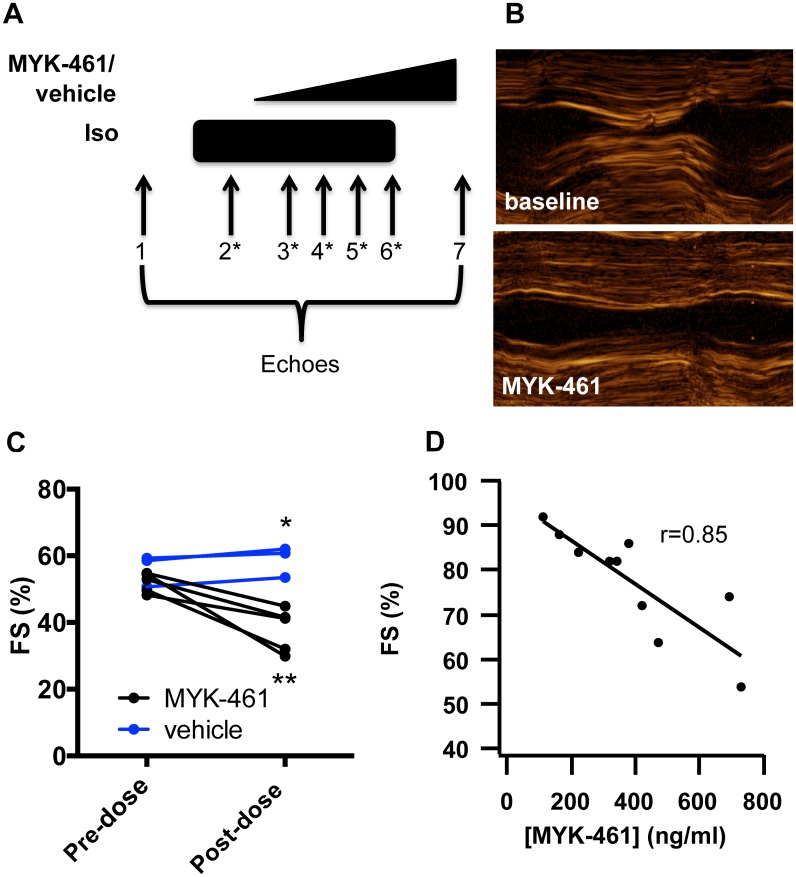
MYK-461 reduces contractility in feline HCM in an exposure-dependent manner. **(A)** Study scheme depicting timing of echocardiograms (numbered) and administration of isoproterenol (iso) at constant dose and MYK-461 or vehicle as a ramp infusion. Timepoints with asterisks correspond to blood draws to measure plasma concentration of MYK-461. Timepoint 1: baseline following anesthesia; timepoint 2: stable isoproterenol; timepoints 3–6: stable isoproterenol with increasing MYK-461 dose; timepoint 7: stable MYK-461 dose without isoproterenol. **(B)** Decrease in contractility with MYK-461 treatment by M-mode echocardiography. **(C)** Before and after plot of FS in response to treatment with MYK-461 (n = 5) or vehicle (n = 3) in the absence of isoproterenol (*, p = 0.03 vs vehicle by unpaired t-test; **, p = 0.01 vs. pre-dose by paired t-test). **(D)** Linear correlation of FS with MYK-461 plasma concentration for timepoints 2–6 in the presence of isoproterenol (n = 3 cats, p<0.0001).

We first compared FS prior to treatment (Fig 2A, timepoint #1) with that on only MYK-461/vehicle (timepoint #7) (Fig 2A). Treatment with MYK-461 reduced FS from 52 ± 3% to 38 ± 7% (Fig 2B and 2C; p = 0.003 vs. vehicle, p = 0.01 vs. pre-treatment). The reduction in FS corresponded to an increase in ventricular diameter at end-systole (0.67 cm vs 0.82 cm, p = 0.03) with a stable end-diastolic diameter (1.40 cm vs. 1.34 cm, p = NS). To determine if MYK-461 can reduce contractility in the presence of adrenergic stimulation, we compared FS in cats receiving only isoproterenol 0.04 μg/kg/min IV (timepoint #2) with FS on both isoproterenol 0.04 μg/kg/min IV and MYK-461/vehicle IV. Treatment with MYK-461 reduced FS from 81 ± 7% to 60 ± 13%, corresponding to a relative reduction of 25% (Fig 2D; p = 0.007 vs. pre-461). To better define the exposure/response relationship, we measured FS and MYK-461 plasma concentrations at three additional times during the MYK-461 infusion (timepoints #3–5, S2B Fig). Across all measurements there was a linear correlation between FS and MYK-461 plasma concentrations (Pearson’s r = 0.85; p<0.0001; Fig 2D and S1B Fig) with each 100 ng/ml increase in MYK-461 concentration lowering FS by 4.9%. These data show that MYK-461 reduces contractility in cats with HCM, and can do so in the presence of adrenergic stimulation.

**Fig 3:**
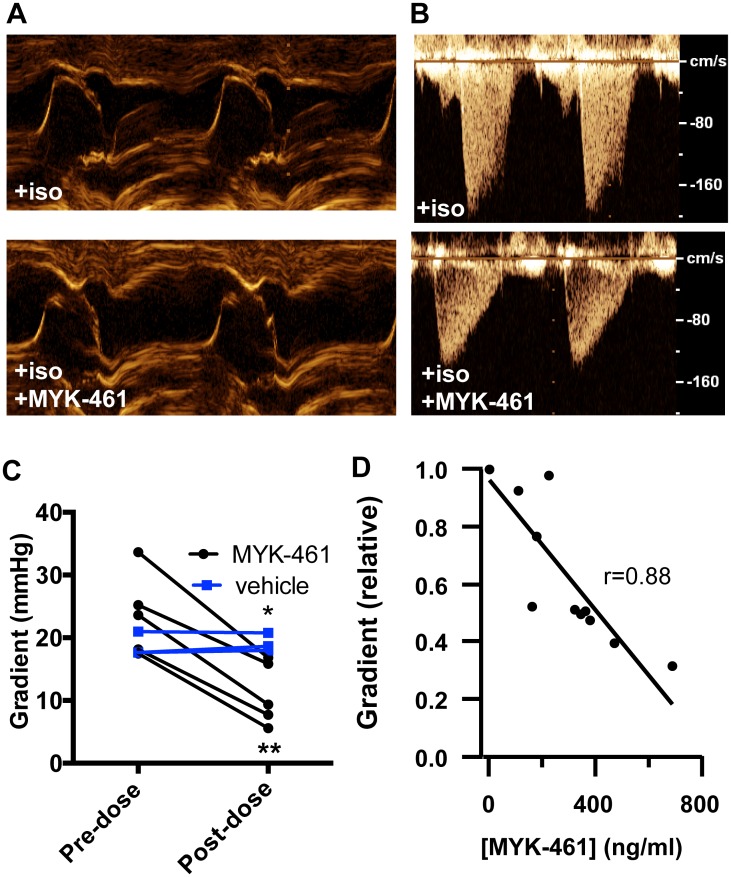
Treatment with MYK-461 abolishes mitral valve SAM and reduces LVOT gradients in cats with HCM. Treatment with MYK-461 abolishes **(A)** SAM and **(B)** LVOT obstruction induced by isoproterenol treatment**. (C)** Before and after plot of LVOT pressure gradient for cats treated with isoproterenol 0.04 μg/kg/min IV and either MYK-461 (n = 5) or vehicle (n = 3) (*, p = 0.038 vs. vehicle by unpaired t-test; **, p = 0.0007 vs. pre-dose by paired t-test). **(D)** Linear correlation of relative LVOT pressure gradient (normalized to values prior to MYK-461 treatment) with the plasma concentration of MYK-461 at timepoints 2–6 (n = 3 cats, p<0.0001).

We next investigated the effect of MYK-461 on provoked LVOT pressure gradients by echocardiography. We compared hemodynamics in cats on isoproterenol alone (timepoint #2) to cats on both isoproterenol and MYK-461 (timepoint #6). Treatment with MYK-461 eliminated SAM in 5/5 subjects, whereas SAM persisted in 3/3 subjects treated with vehicle alone (Fig 3A; p = 0.018). Pressure gradients across the LVOT dropped to 11.1 ± 5.0 mmHg with MYK-461 treatment, whereas vehicle treated cats maintained stable LVOT pressure gradients (Fig 3B and 3C; p = 0.038 vs. vehicle, p = 0.0007 vs. pre-treatment). Despite the reduction in contractility and LVOT pressure gradient with MYK-461, systolic blood pressure increased a modest amount from 96 ± 9 mmHg to 108 ± 8 mmHg (S3 Fig), suggesting a hemodynamic benefit of relieving obstruction. To define the exposure/response relationship for LVOT pressure gradient with MYK-461, we measured maximal pressure gradients in the LVOT and MYK-461 plasma concentrations at three additional times during the MYK-461infusion (timepoints #3–5). The relative pressure gradient across the LVOT correlated linearly with MYK-461 plasma exposures (r = 0.88; p<0.0001; Fig 3D), and each 100 ng/ml increase in MYK-461 concentration reduced the gradient by 11% (2.3 mmHg). These data demonstrate that specific reduction in contractility by selectively targeting myosin with MYK-461 can relieve dynamic LVOT pressure gradients induced by adrenergic stimulation in cats with HCM in an exposure-dependent manner.

## Discussion

In this study, we identified a subset of cats from a research colony predisposed to HCM that exhibit SAM and LVOT obstruction at rest. In detailed echocardiographic studies under anesthesia, SAM and LVOT obstruction could be provoked by adrenergic stimulation. MYK-461, a small molecule that inhibits sarcomere contractility by selectively targeting myosin, reduced FS in cats without affecting heart rate, and this was sufficient to acutely eliminate SAM and relieve LVOT obstruction. These findings provide the first proof of principle that provokable SAM and LVOT obstruction can be reduced by selectively reducing contractility using a novel mechanistic class of direct sarcomere inhibitors. Because other available negative inotropes have negative chronotropic effects (i.e. beta blockers and calcium channel blockers) or vasoactivity (disopyramide), these data provide direct evidence that reduction in contractility is sufficient to relieve LVOT obstruction.

Our data suggest that cats with HCM may serve as a model for provokable LVOT obstruction. Mouse models have been invaluable for characterizing the genetics and cellular pathology of HCM but have been limited by differences in physiology from human disease, most prominently the absence of LVOT obstruction. Resting obstruction caused by SAM is a feature of feline HCM in client-owned cats as well as in this research colony [17]. Because of the hemodynamic effects of anesthesia, additional provocation was required to elicit LVOT obstruction in this study. Isoproterenol was chosen because of its well-established property of inducing gradients in HCM [24]. Although isoproterenol reliably augments LVOT obstruction in HCM, previous studies in animals [25] and humans [26] have shown that it does not induce SAM and LVOT outflow gradients in the normal ventricle. This suggests that isoproterenol stimulation in cats with HCM and dynamic obstruction represents a distinct animal model to understand and develop therapeutics for this disease pathophysiology.

Identification and treatment of LVOT obstruction is a cornerstone of therapy for patients with HCM and symptoms of dyspnea and/or angina. The etiology of these symptoms is incompletely understood, but they are thought to arise from a combination of interrelated physiologic abnormalities including outflow obstruction, ischemia, diastolic dysfunction and mitral regurgitation [27]. Current treatment guidelines recommend initiation of medicines (beta blockers and calcium channel blockers) that can reduce heart rate and/or inotropy to alleviate obstruction and reduce myocardial oxygen demand [28]. Despite the physiologic rationale for these treatments, they have not shown clear benefit on symptoms or outcomes in randomized trials [29]. Indeed, in one single center study of 509 patients with obstructive HCM, 299 (58.7%) had persistent symptoms despite treatment with beta blockers and/or calcium channel blockers [30]. These data suggest that non-selective negative inotropy and chronotropy alone may not be sufficient to address the symptoms of HCM with LVOT obstruction.

A more definitive option for patients with symptomatic, obstructive HCM despite medical therapy is septal reduction by surgical excision or alcohol ablation. Widening of the LVOT with these interventions reduces LVOT pressure gradients with low procedural mortality and morbidity, particularly with contemporary surgical techniques at high-volume centers [31]. Non-randomized, retrospective studies suggest that many patients have improved symptoms and functional capacity as well as a potential improvement in long-term mortality [9,32]. However, a recent meta-analysis shows that symptomatic improvement is not uniform, with up to 50% of patients not experiencing an improvement in NYHA functional class after septal reduction [33]. This residual symptom burden suggests that eliminating the gradient alone may not be sufficient to treat the disease. The molecular pathophysiology of HCM—which ultimately gives rise to LVOT obstruction—also produces other abnormalities such as fibrosis and diastolic dysfunction that are important sources of morbidity not addressed by septal reduction [34].

Increasing understanding of the genetic basis of HCM has defined it as a disease of the sarcomere. Mutations in genes encoding sarcomere proteins produce molecular biophysical abnormalities that ultimately lead to the contractile dysfunction of the heart, fibrosis, hypertrophy and other downstream pathologies [18]. Based on these molecular insights, MYK-461 was discovered in a screen for direct inhibitors of sarcomere activity [19]. Unlike beta blockers or calcium channel blockers, sarcomere modulators act directly at the motor proteins to counteract the biochemical defect in HCM without affecting calcium handling or other aspects of myocyte function. Sarcomere inhibitors could thus offer a targeted therapy for HCM that more specifically addresses its underlying abnormalities. Furthermore, because perturbations in motor function precipitate other manifestations of HCM (fibrosis, myofibrillar disarray, arrhythmias), correcting the biochemical defect could also modify the cellular and molecular pathology of disease.

Our study has several limitations. All studies of compound action were performed under anesthesia, which alters multiple aspects of cardiovascular physiology, including contractility, heart rate and vascular tone. Because of these changes, provocation by isoproterenol was necessary to induce sustained LVOT pressure gradients despite the presence of LVOT obstruction in conscious cats. Nevertheless, the pattern of SAM and LVOT obstruction under these conditions was consistent with that found in conscious cats as well as in humans, suggesting that the induced gradients could be a valid model to study the effects of modulating contractility. This model may best reflect the LVOT gradients induced upon exercise provocation, which are present in approximately one in three human HCM patients.

Thus, our studies provide proof of principle that a sarcomere inhibitor can acutely relieve provocable LVOT gradients in cats with HCM. These data pave the way for chronic studies—in animals and humans—under more physiologic conditions to test the durability of response and the appearance of salutary remodeling and disease modification. Our results highlight the value of companion animals in translational research and raise the potential of a novel therapeutic approach in HCM patients with LVOT obstruction.

## Supporting Information

S1 Fig Heart rate and blood pressure for cats on isoproterenol and treated with MYK-461 or vehicle. Treatment with MYK-461 does not alter heart rate but increases systolic blood pressure (SBP) in cats on a stable dose of isoproterenol (p = 0.004), whereas SBP does not change with vehicle treatment.
(EPS)

S2 Fig LVOT obstruction in a conscious cat with HCM. **(A)** Parasternal long axis image with color Doppler in a conscious cat with HCM show acceleration of flow in the LVOT. (**B)** Pulse wave Doppler profiles from the parasternal long axis view in a conscious cat with HCM demonstrate elevated velocities in the LVOT (scale bar = 1 m/s).
(EPS)

S3 Fig MYK-461 administration schedule and plasma concentrations in treated cats. **(A)** Ramp infusion protocol for IV administration of MYK-461 **(B)** Plasma concentrations for cats treated with MYK-461 (n = 3).
(EPS)

## Abbreviations

FS: fractional shortening
HCM: Hypertrophic cardiomyopathy
LVOT: Left ventricular outflow tract
SAM: systolic anterior motion
